# I-Kaz methodology for predicting tool life of AlCrN—Coated WC—Co inserts in the machining of AISI 304 steel

**DOI:** 10.1016/j.mex.2025.103706

**Published:** 2025-11-04

**Authors:** Mogana Priya Chinnasamy, Rajasekar Rathanasamy, Swetha R Kumar, Sathish Kumar Palaniappan

**Affiliations:** a School of Mechanical Engineering, Vellore Institute of Technology, Chennai campus, Chennai 600 127, India; b Department of Mechanical Engineering, Kongu Engineering College, Erode, Tamilnadu 638 060, India; c School of Electrical Engineering, Vellore Institute of Technology, Chennai Campus, Chennai 600 127, India; d Natural Composites Research Group Lab, Department of Materials and Production Engineering, The Sirindhorn International Thai-German Graduate School of Engineering, King Mongkut’s University of Technology North Bangkok, Bangsue 10800, Thailand

**Keywords:** Stainless Steel 304, Taguchi analysis, Microflown sensor, I-Kaz Coefficient

## Abstract

This study proposes a real-time tool wear monitoring approach for dry milling of AISI 304 stainless steel using a Microflown PU sensor and the I-Kaz™ statistical feature. A Taguchi L18 orthogonal array was adopted to optimize cutting speed, feed rate, depth of cut and tool type (uncoated and AlCrN-coated WC–Co inserts) based on surface roughness and flank wear responses. ANOVA revealed that tool type was the most significant factor, contributing approximately 85 % to flank wear variation, followed by depth of cut and cutting speed. The optimal combination: AlCrN-coated insert, cutting speed of 1250 rpm, depth of cut of 0.50 mm and feed rate of 0.04 mm/rev minimized both responses. The proposed I-Kaz-based monitoring approach established a strong inverse power-law relationship between the I-Kaz coefficient and flank wear Z_2_^α^ =*a*(VB)^-n^, achieving R^2^>0.96, indicating high accuracy and stability across repetitions.

• This study introduces the first application of a Microflown PU sensor for near-field acoustic monitoring in milling operations.
• The I-Kaz™ feature is demonstrated as a computationally efficient and accurate method for real-time tool wear prediction.
• The research integrates cutting parameter optimization and predictive monitoring within a single experimental framework.

## Graphical abstract

**Figure:**
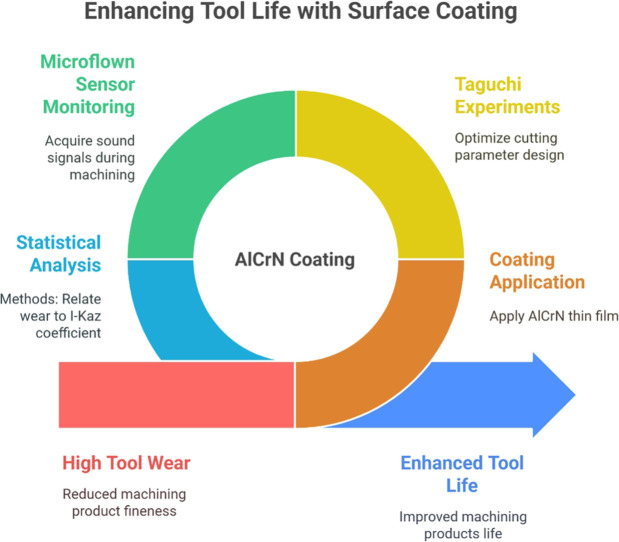

**Specifications table**

**Table:** 

**Subject area**	Engineering
**More specific subject area**	Manufacturing
**Name of your method**	I-Kaz method
**Name and reference of original method**	Ahmad, Muhamad Arif Fadli, et al. "Development of tool wear machining monitoring using novel statistical analysis method, I-kaz™." Procedia Engineering 101 (2015): 355–362.
**Resource availability**	Nil

## Background

In recent years, manufacturing has involved substantial metal cutting operations. There are pre-planned and better-quality processes, which need to reach the desired output or reduce production costs in a machining operation [1]. Most of the researchers have a challenge to meet accurate quality machining and productivity performance intended to continuous monitoring of efficient operations [2]. For machining in various levels, cutting inserts are one of the most needed components. In accordance to avoid downtime operation due to failures of cutting inserts, there is a necessity to develop advanced technology that leads to achieve superior products and higher productivity [3,4]. The usage of lubricants or fluids for various levels of cutting operations are more expensive approximately 7 to 17 percentage of labor cost. Moreover, these fluids are connected with numerous complications tackled in operating atmosphere and also difficulties even in the removal of wastage [5]. The lubrication method leads to more initial price, service, wastage of cutting fluids and environmental issues due to negative effects of fluids and presence of chemical composition [6]. To avoid these kind of environmental circumstances, researchers suggested dry machining operation strategies which helps to enhance the preventive measures of environment [7].

One of the most widely used machining operations is the milling process in which surface machining influenced on process cutting parameters and selection of cutting tool inserts. Surface roughness indicates quality of the surface elements based on corrosion, wear resistance and material fatigue strength. In addition to that quality of machined product are decided by the tool inserts like as uncoated and coated inserts [8,9]. Many researchers suggested that there is a drop of energy consumption in a last few decades due to unsuitable selection of cutting process parameters, cutting tool inserts and optimization path [10]. In last few decades, coating inserts are actively reached among the researchers for application purposes. Tool life of cutting tool inserts has improved slightly ten times due to hard coating process and it reduces the insert wear rate [5,11]. In selection of appropriate tool for exact workpiece is a major risk as it consumes less energy and tool life should be better. In advanced development of industries especially hard steels machining, which creates place for high demand inserts [5,12]. Suresh et al. investigated the efficiency of carbide inserts, which enhanced while machining of AISI-4340 steel material with cathodic vapor deposition coated inserts [13]. In 1980s most of the scientists suggested marketable PVD coatings is TiN for HSS drill applications. Owing to obtain desirable objectives, numerous applications of coatings has been identified to meet easier removal of materials on cutting inserts [14]. Beake et al. have been reported that AlCrN and AlTiN coated inserts achieved a better performance even at extreme conditions since as in a higher temperatures it has a decent characteristics of oxidation resistance, hot hardness and wear resistance [15]. The author experimentally investigated as much higher abrasive and wear impact occurred in a AlTiN and AlCrN when compared with CrN layers at raised temperatures. Some other researchers conducted experiments on AlCrN, AlTiN, AlCrNbN of PVD coating technique discovered that superior wear resistance at a surrounding temperature of about 600 °C [14,16].

Most of the studies explained that operating parameters at different cutting tool and conditions are optimized using different techniques such as surface methodology, experimental designs and statistical analysis [[17], [18], [19]]. Rocha et al. applied response surface methodology to optimize various process parameters for increasing level of tool life, and surface roughness is lesser with PCBN wiper inserts on AISI H13 tool. Taguchi is used to predict the optimal parameters to raise MRR, reduction of roughness and minimal energy consume through optimization of cutting parameters [5,20]. There are extensively used RSM technique to optimize various cutting parameters because of their effective method [6]. The authors Gunay and Yuce developed Taguchi method of different cutting parameters in turning for surface roughness optimization. L18 orthogonal array has been developed to measure the optimum conditions by means of signal to noise ratio method on “smaller the better” technique [21].

Commonly TCMs need diagnostics and prognostics methods which include tool health in different states of state and evaluating machine life respectively. Two techniques are available to detect the state of wear namely, direct and indirect. Direct techniques help to detect state of wear through offline mode. Indirect methods are measured through online mode where proper signals via sensors are converted into flank wear [3]. In a recent time, Ambhore et al. suggested several innovative developments on TCMs, in such methods of offline where exposed to lesser accuracy comparatively with online modes as an outcome is defective. Four elements involved in TCMs are sensor operations, data acquisition, feature extraction and appropriate techniques [22].

The relation of tool wear and acquired signals are developed in terms of statistical method by using I-Kaz coefficient, there is no interventions takes place during machining operations [23]. Microphone sensor helps to acquire the sound signals whereas identify wear endlessly without disconnecting inserts and workpiece from holder [24]. Tool condition monitoring with very near field acoustic sensor (Microflown sensor) is not found elsewhere in the literature. This work focuses on optimization of machining parameters to enhance tool life and design of tool condition monitoring system with I-Kaz coefficients.

## Method details

**Fig. 1:**
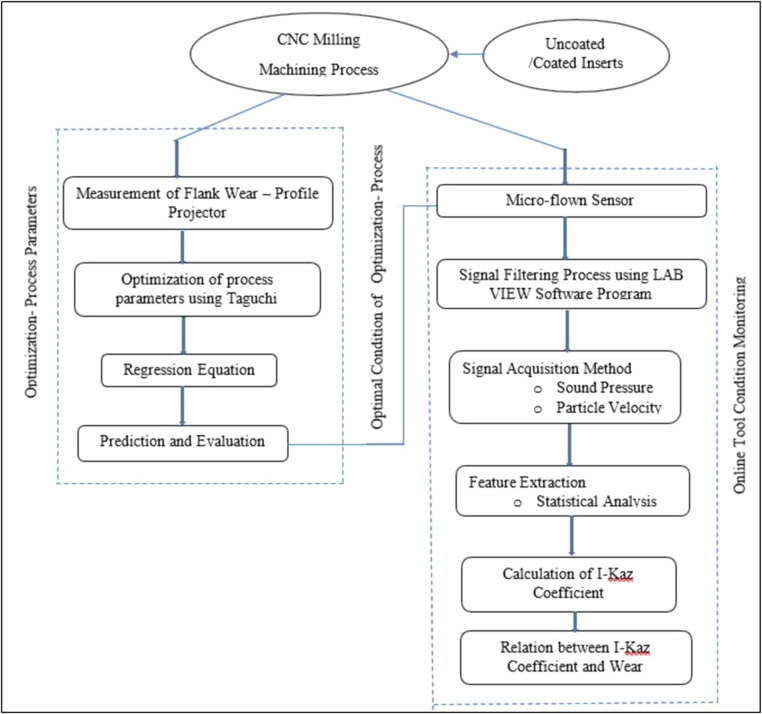
Proposed Research Methodology.

The proposed research method is shown in Fig. 1. The cutting inserts selected for this machining were uncoated and coated inserts. The marketable grade of uncoated carbide tool inserts (XDHT090308-THM) was procured from Widia Pvt. Ltd. and AlCrN coating was deposited on uncoated insert through physical vapour deposition from Oerlikon Balzer’s industrial coating system.

In this research study, stainless steel (AISI 304) was used as workpiece with a length of 180 mm and a diameter of 70 mm. AISI 304 steel is widely applied in various manufacturing industries such as pipes, flanges, bar, sheets, etc. because of their good corrosive resistance and oxidation resistance. The elemental chemical composition of AISI-304 in terms of weight percentage is listed in Table 1.

**Table 1:** Elemental Chemical Composition of AISI 304.

Element	Fe	Cr	Ni	Mn	Si	C	P	N	S
Weight %	Balance	18.15	8.50	1.38	0.37	0.06	0.03	0.005	0.013

The experiments were performed in CNC l-Mill 55 with a maximum run of 6000 rpm and 11 kW of input power. The uncoated carbide and AlCrN coated inserts were engaged to perform the machining operations under dry environment. M680 tool holder was mounted to hold cutting tool inserts. The levels of process parameters were accounted based on literature studies and recommended handbook. The cutting parameters are listed in Table 2. Based on the prescribed parameters, L_18_ orthogonal array were structured through Taguchi design of experiments. Signal to noise ratio characteristics is used to optimize final desired outcome of tool wear and surface roughness responses at ‘smaller the better ratio’.

**Table 2:** Cutting parameter conditions.

Workpiece Material	: AISI 304		
Medium of coolant	: No coolant – Dry Environment		
Machining Time	: 10mins		
Coating	: Uncoated carbide & AlCrN coated carbide Inserts		
Factors	Level 1	Level 2	Level 3
Spindle speed	750	1000	1250
Feed rate	0.02	0.04	0.06
Depth of cut	0.5	0.75	1
Output Responses	: Surface roughness and Flank wear		

### Measurement of flank wear and surface roughness

The tool wear rate is determined during each iteration of the experiments by employing a profile projector to assess the condition of the tool. The mean of three data points has been recorded and presented in Table 3. The Mitytyo surface roughness tester was utilized to determine roughness values at three different locations. The resulting numerical values representing the average roughness of the machined work surface were recorded and presented in Table 3.

**Table 3:** Experimental data of surface roughness and flank wear with S/N ratio values.

S.No.	Tool	Cutting Speed	Depth of Cut	Feed Rate	Surface Roughness (µm)	Tool Wear (mm)	S/N Ratio of Surface Roughness	S/N Ratio of flank wear
1	Uncoated	750	0.50	0.02	0.1280	0.0330	17.8558	29.6297
2	Uncoated	750	0.75	0.04	0.1390	0.0340	17.1397	29.3704
3	Uncoated	750	1.00	0.06	0.1380	0.0350	17.2024	29.1186
4	Uncoated	1000	0.50	0.02	0.0810	0.0350	21.8303	29.1186
5	Uncoated	1000	0.75	0.04	0.0840	0.0340	21.5144	29.3704
6	Uncoated	1000	1.00	0.06	0.1420	0.0400	16.9542	27.9588
7	Uncoated	1250	0.50	0.04	0.0406	0.0360	27.8295	28.8739
8	Uncoated	1250	0.75	0.06	0.0780	0.0400	22.1581	27.9588
9	Uncoated	1250	1.00	0.02	0.0812	0.0430	21.8089	27.3306
10	AlCrN	750	0.50	0.04	0.1160	0.0130	18.7108	37.7211
11	AlCrN	750	0.75	0.06	0.0119	0.0150	38.4891	36.4782
12	AlCrN	750	1.00	0.02	0.1200	0.0200	18.4164	33.9794
13	AlCrN	1000	0.50	0.04	0.1210	0.0140	18.3443	37.0774
14	AlCrN	1000	0.75	0.06	0.1220	0.0160	18.2728	35.9176
15	AlCrN	1000	1.00	0.02	0.1210	0.0250	18.3443	32.0412
16	AlCrN	1250	0.50	0.06	0.1230	0.0150	18.2019	36.4782
17	AlCrN	1250	0.75	0.02	0.1230	0.0199	18.2019	34.0229
18	AlCrN	1250	1.00	0.04	0.1250	0.0250	18.0618	32.0412

### Development model of continuous online tool monitoring

In this study, the sound produced during the machining process was captured using a state-of-the-art sound sensor known as the Microflown-PU sensor. The description of the sensor's specifications is as follows.

•Model : MFPA2–90,130 Diameter: 12.7mm

•Sensitivity: 65mV/Pa – sound pressure Frequency: 40 to 80 kHz

**Fig. 2:**
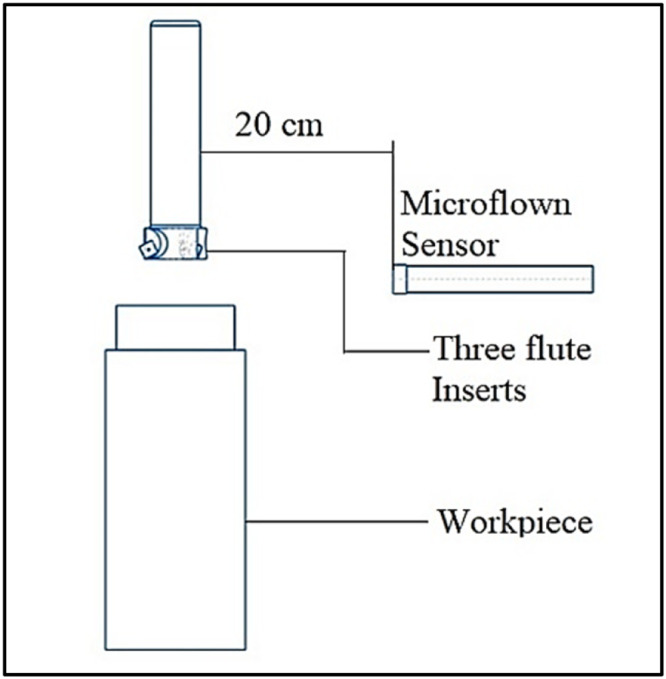
Placement of Sensor.

**Fig. 3:**
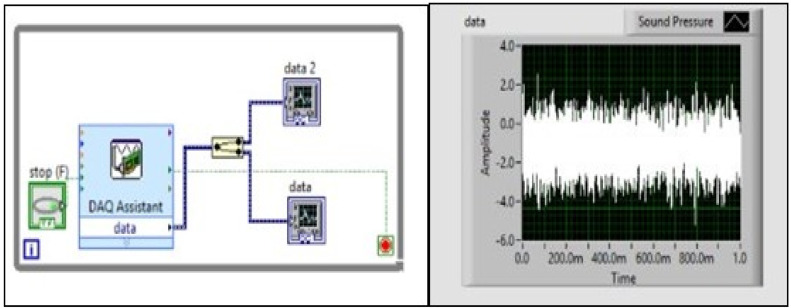
Data Acquisition Processing Signals.

**Fig. 4:**
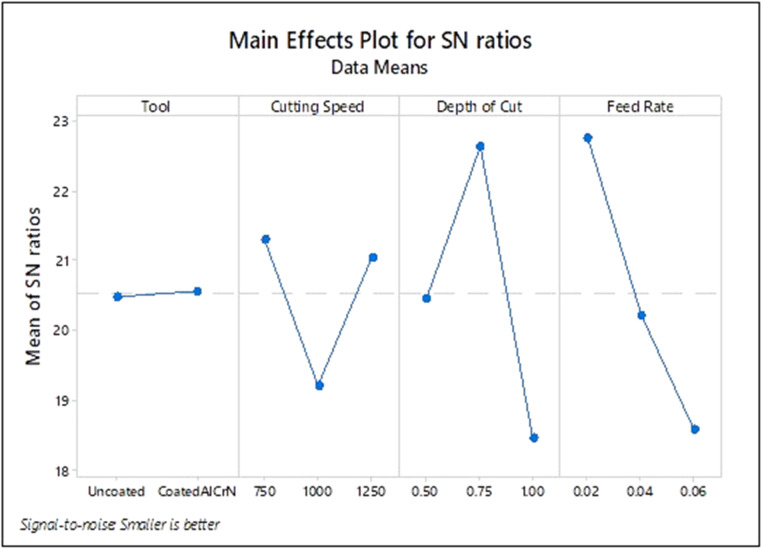
Input parameters for S/N plot – Surface Roughness.

Fig. 2 illustrates the fundamental principle underlying the process of sound pressure measurement. Measuring particle velocity and sound pressure during the machining process is deemed appropriate, as it enables the considerable differentiation of signals between tools that are functioning well and those that are erroneous. The data obtained from the microflown sensor was collected under controlled dry conditions during the experiments, with all external noise sources effectively eliminated. To accurately capture sound pressure signals without interference and record the corresponding data, it is necessary to utilize the NI c DAQ – 9174 card. Fig. 2 illustrates the positioning of the sensor within the experimental configuration. The process of acquiring data from the sensor was carried out using NI LabVIEW, as depicted in Fig. 3. Fig. 4 illustrates the LabVIEW application utilized for the purpose of acquiring data in the context of frequency response analysis. The signals obtained were subjected to statistical analysis using measures such as kurtosis and I-Kaz coefficient. A correlation was established between the I-Kaz coefficient and flank wear. The wear of cutting inserts can be continually measured without detaching them from the machine by utilizing statistical analysis, namely the I-Kaz coefficient.

Nuwai established the foundation for the I-Kaz methodology, which focused on the examination of random or non-deterministic characteristics of signals [25] The frequent utilization of the r_th_ order moment M_r_ is common in the categorization of deterministic features. The r_th_ order moment, M_r_, for the discrete signal in the frequency band can be mathematically represented as follows:

(1) $$M_{r}=\frac{1}{N}\sum_{i=1}^{n}{\left(x_{i}-\;\bar{x}\right)}^{r}$$

where, N - Number of data points, $X_{i}$- Data at instantaneous points, $\bar{X}$- Mean.

In the first stage, signals were acquired through the utilization of LAB-View. A set of n values was obtained by measuring both sound pressure and particle velocity in combination. The kurtosis coefficient K for discrete data can be mathematically represented as follows:Initially, signals are obtained using LAB-View, n number of values by a combination of sound pressure and particle velocity were gained. The kurtosis K for discrete data can be expressed as:

(2) $$K=\frac{1}{N\sigma^{4}}\sum_{i=1}^{n}{\left(x_{i}-\bar{x}\right)}^{4}$$

where σ -Variance.

The I-Kaz coefficient is a mathematical measure used to quantify the dispersion of data distribution by calculating the distance between each data point and the centroid of the signal. The coefficient denoted as I-Kaz will be defined as follows:

(3) $$I-Kaz\;2D\;Coef=\;\sqrt{\frac{1}{N}\;\left(M^{I}\right)+\frac{1}{N}\;\left(M^{II}\right)}$$

where, M^I^ and M^II^ - the moment in the channel I and II

By substituting Eqs. (1), 2 in 3, I- Kaz coefficient can be formulated as depicted in Eq. (4) and it is denoted by the symbol $Z_{2}^{\infty }$.

(4) $$Z_{2}^{\infty }=\;\frac{1}{N}\sqrt{K_{I}S^{I}+K_{II}S^{II}}$$

where, $K_{I}$ and $K_{II}$- Kurtosis of signal in ch-I and ch-II

$S^{I}\;$and $S^{II}$- Standard deviation of signal in ch-I and ch-II [23]

Fig. 3 shows the data acquisition processing signals through Lab-View program in a time domain response. Then I-Kaz coefficient can be evaluated from observed signals.

### Optimization of process parameters

The research experimental tests were determined based on L_18_ orthogonal array in a CNC milling machining operation. For all L_18_ (OA) experiments, flank wear and surface roughness were recorded and listed in Table 3. Smaller Signal to Noise ratio is chosen to examine best suited condition where lesser flank wear and surface roughness of product is attained to perform superior milling operation [26]. The signal to noise ratio is defined as

S/N ratio = −10 log10 (mean of sum of squares of measured-ideal)

S/N ratio was estimated using Minitab–Taguchi method and data depicted in Table 3. Then optimal conditional parameters were identified by choosing the largest S/N ratio.

Fig. 4 shows that optimal cutting parameter of surface roughness during milling machining operation under dry environment of SS 304 when conditions of tool: Coated AlCrN; spindle speed: 750 rpm; depth of cut: 0.75 mm; feed rate: 0.02 mm/ rev.

**Fig. 5:**
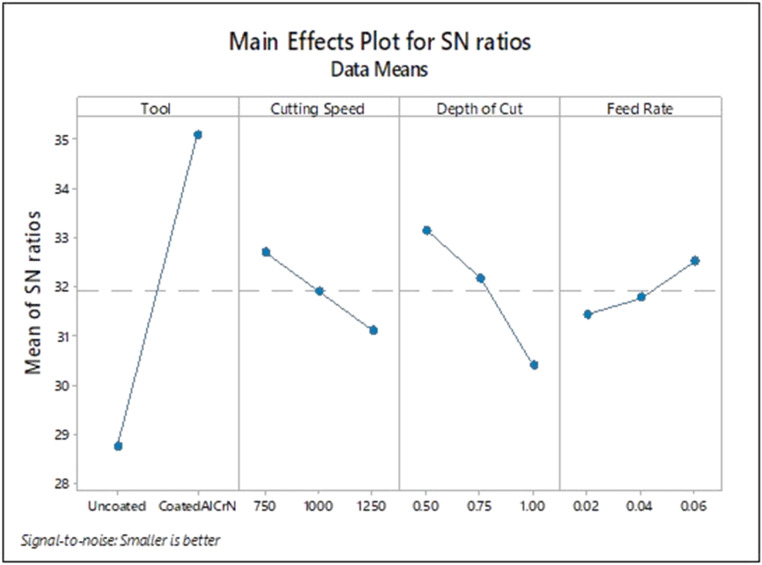
Input parameters for S/N plot – Wear.

From Fig. 5, coated AlCrN; spindle speed:750 rpm; depth of cut: 0.5 mm and feed rate: 0.06 mm/rev were obtained as the best optimal cutting parameters for tool wear.

To evaluate the influence of individual machining parameters on flank wear, an analysis of variance (ANOVA) based on the Taguchi method was performed, as summarized in Table 4. The ANOVA results quantify the relative effect of each process parameter on the output response, while the percentage contribution ( %C) provides a clear indication of the dominant factors affecting tool wear [24]. Additionally, 95 % confidence intervals have been included to provide statistical reliability for each estimated effect, ensuring the robustness of the model. From the ANOVA results, it is evident that the tool type has the most significant impact on flank wear, contributing 85.41 % to the total variation. This clearly indicates that the selection of tool insert material (coated vs. uncoated) plays a dominant role in determining tool life and wear behavior. Specifically, the uncoated insert exhibits a higher wear rate, whereas the AlCrN-coated insert maintains a significantly lower wear rate, confirming its superior wear resistance under the tested cutting conditions. The depth of cut is the second most influential factor, contributing 8.09 % to the overall variation. Increasing the depth of cut increases the contact area and stress at the tool–workpiece interface, leading to higher flank wear. The cutting speed shows a moderate influence with 3.85 % contribution, reflecting the fact that higher cutting speeds elevate the temperature at the tool–chip interface, accelerating wear. Finally, feed rate has a minimal effect (0.66 %), indicating that within the selected range, variations in feed rate do not significantly alter the flank wear rate.

**Table 4:** Analysis of Variance.

Source	DF	Adj SS	Adj MS	F-Value	P-Value	% Contribution	95 % Confidence Interval
Regression	4	0.001780	0.000445	160.14	0.000		
Cutting Speed	1	0.000070	0.000070	25.05	0.000	3.85	0.000070 ± 0.000012
Depth of Cut	1	0.000147	0.000147	52.91	0.000	8.09	0.000147 ± 0.000015
Feed Rate	1	0.000012	0.000012	4.25	0.040	0.66	0.000012 ± 0.000010
Tool	1	0.001551	0.001551	558.34	0.000	85.41	0.001551 ± 0.000042
Error	13	0.000036	0.000003				
Total	17	0.001816					

**Fig. 6:**
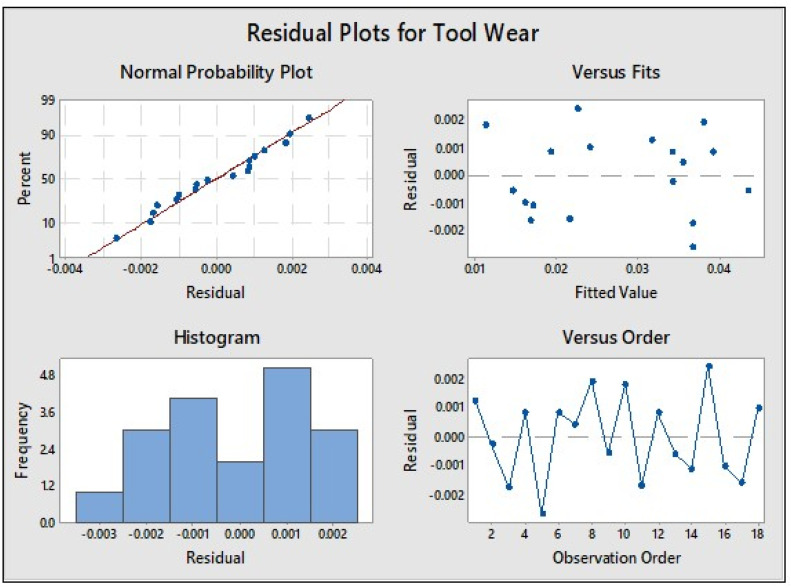
Normal Probability and Residual plots.

The p-values for all parameters are <0.05, confirming the statistical significance of each factor and validating the reliability of the model. The residuals have been plotted (Fig. 6) to further confirm that the assumptions of normality, constant variance and independence are satisfied, ensuring the adequacy of the regression model. The high model fit, with a coefficient of determination R^2^=98.01 % (Table 5), indicates that the developed regression equation (Table 6) accurately captures the relationship between machining parameters and flank wear. Overall, the analysis demonstrates that tool material selection is the key determinant of flank wear, followed by depth of cut and cutting speed, while feed rate has a negligible effect. The inclusion of confidence intervals provides an added measure of statistical reliability, supporting the robustness and predictive capability of the regression model for optimizing tool life.

**Table 5:** Model Summary.

S	R-sq	R-sq (adj)	R-sq (pred)
0.0016668	98.01 %	97.40 %	96.20 %

**Table 6:** Regression Equation.

Tool			
Uncoated	Tool Wear	=	0.01852 + 0.000010 Cutting Speed + 0.01400 Depth of Cut - 0.0496 Feed Rate
Coated AlCrN	Tool Wear	=	−0.00005 + 0.000010 Cutting Speed + 0.01400 Depth of Cut - 0.0496 Feed Rate

Fig. 6 presents the normal probability and residual plots. The normal probability plot indicates that the residuals closely follow the inclined reference line, confirming that they are approximately normally distributed. The residuals exhibit a uniform distribution, suggesting no systematic bias in the model. The plot of fitted values versus residuals shows that the residuals are scattered evenly across the range, with no clustering observed in any region, indicating homoscedasticity. The residuals are minimal in magnitude, ranging between –0.003 and +0.002, which demonstrates the model’s accuracy and the reliability of the predictions.

**Fig. 7:**
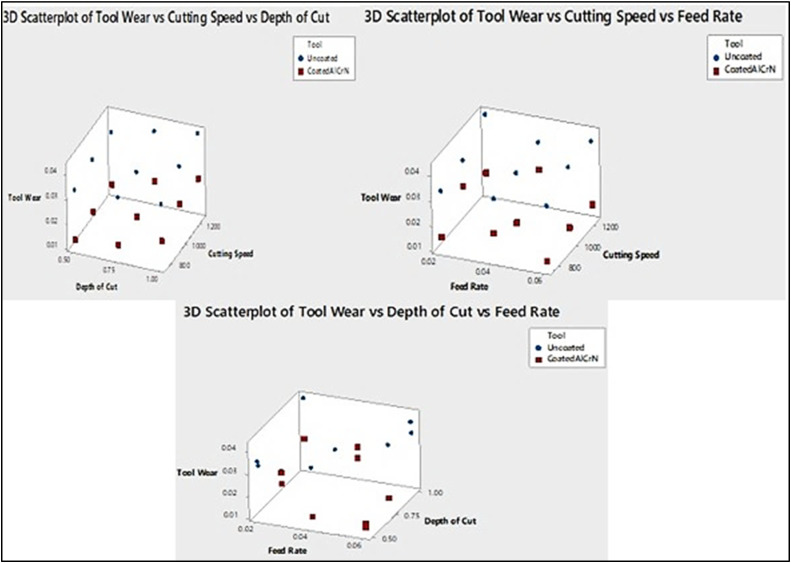
Effect of cutting speed, feed rate and depth of cut on tool wear.

The effects of cutting speed, depth of cut and cutting speed, feed rate and depth of cut, feed rate on the tool wear are displayed in Fig. 7. It revealed that there is a minimum level of flank wear for coated insert when the higher feed rate and lower spindle speed and the same trend is followed in the feed rate and depth of cut and for uncoated insert there is a rapid increase of wear rate at high level of spindle speed and depth of cut.

## Method validation

A LabVIEW platform was utilized to analyze the acquired sound pressure and velocity signals in the time-domain response during the CNC milling of AISI 304 stainless steel. The signals were captured using a Microflown sensor connected to a high-precision data acquisition system. The I-Kaz coefficient Z_2_^α^ was calculated using Eq. (4), which statistically characterizes the vibrational signals in the milling process. The acquired signals were recorded continuously over a milling interval of 10 min, generating a total of 70,000 cycles, with Z_2_^α^ measured at every 10,000-cycle interval using the programmed software.

**Fig. 8:**
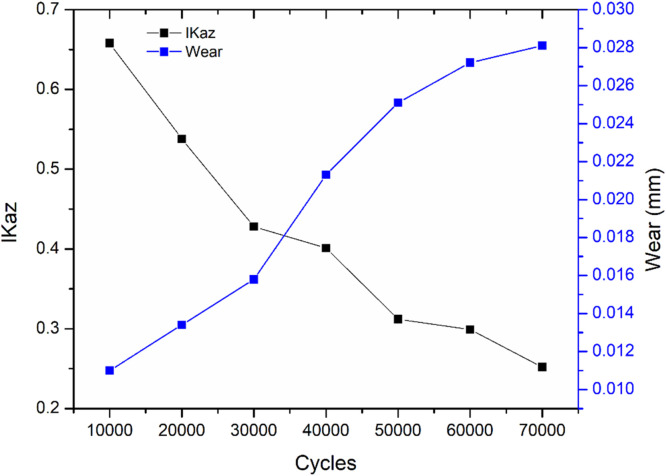
I-Kaz Coefficient Vs Flank wear.

For optimized process parameters determined through the Taguchi method, the relationship between flank wear (VB) and the I-Kaz coefficient was evaluated. As illustrated in Fig. 8, an increase in the number of cycles results in a corresponding rise in flank wear, while the I-Kaz coefficient shows a decreasing trend. Initially, at 10,000 cycles, the flank wear is minimal (∼0.011 mm), and the I-Kaz coefficient is relatively high (∼0.65), indicating lower energy dissipation due to minimal tool wear. As the milling progresses to 70,000 cycles, the flank wear increases (∼0.028 mm), while the I-Kaz coefficient drops to ∼0.25, reflecting increased energy absorption and damping due to progressive tool degradation.

The relationship between I-Kaz coefficient and flank wear can be mathematically expressed using a power-law model:

(5) Z_2_^α^ =*a*(VB)^-n^

where a and n are constants dependent on cutting parameters, and VBV_BVB​ represents the measured flank wear. This model indicates that the I-Kaz coefficient decreases non-linearly with an increase in flank wear, suggesting that tool degradation significantly affects the vibrational characteristics of the milling process. The trend observed in Fig. 8 demonstrates that the coefficient is sensitive to incremental tool wear, making it a reliable indicator for monitoring tool condition and predicting remaining tool life.

Furthermore, the figure highlights that tool wear accelerates after 40,000 cycles, as evidenced by the steeper increase in flank wear and sharper decline in I-Kaz coefficient. This behavior indicates that initial tool wear progresses gradually, but once a critical wear threshold is reached, the wear rate accelerates, which is consistent with typical tool life behavior in high-speed milling of stainless steel. Such insights provide a quantitative basis for predictive maintenance and optimization of cutting parameters to maximize tool life and maintain surface quality. The above equation is used to determine tool wear during a machining operation. The GUI displays real-time flank wear by monitoring sound pressure. Using the optimized cutting parameters, the system predicts the real-time wear rate through the GUI application.

## Conclusion

Dry milling of AISI 304 stainless steel was carried out using both uncoated and AlCrN-coated WC–Co inserts to analyze tool wear and surface quality under optimized cutting conditions. Taguchi L18 orthogonal array combined with ANOVA revealed that tool type had the most dominant influence on flank wear (≈85 %), followed by depth of cut and cutting speed. The optimal cutting parameters: AlCrN-coated tool, 1250 rpm cutting speed, 0.50 mm depth of cut, and 0.04 mm/rev feed—produced minimum surface roughness and significantly lower wear compared to uncoated tools. A novel real-time monitoring approach was implemented using a Microflown PU sensor to acquire both sound pressure and particle velocity signals from the cutting zone. The I-Kaz™ statistical feature was computed from these signals and demonstrated a strong inverse power-law relationship with flank wear with high predictive accuracy). This correlation enables early detection of tool wear progression without intrusive sensors or complex frequency-domain analysis, ensuring improved process reliability.

The integration of acoustic signal-based monitoring with process optimization presents a robust and computationally efficient solution for smart manufacturing, offering significant potential for industrial applications in predictive maintenance, adaptive control, and improved machining quality.

## Limitations

Not applicable.

## Ethics statements

No human subjects or sensitive data were involved. Anonymous student reflections were used with consent

## Credit author statement

Conceptualization, Methodology, Writing and Reviewing – Mogana Priya Chinnasamy, Rajasekar Rathanasamy, Swetha R Kumar, Sathish Kumar Palaniappan

## Declaration of generative AI and AI-assisted technologies in the writing process

During the preparation of this work the author(s) used Quill bot in order to check language and grammar of this manuscript. After using this tool/service, the author(s) reviewed and edited the content as needed and take(s) full responsibility for the content of the publication.

## Declaration of interests

The authors declare that they have no known competing financial interests or personal relationships that could have appeared to influence the work reported in this paper.

## Data Availability

Data will be made available on request.
